# Comparison of Contaminant Transport in Agricultural Drainage Water and Urban Stormwater Runoff

**DOI:** 10.1371/journal.pone.0167834

**Published:** 2016-12-08

**Authors:** Ehsan Ghane, Andry Z. Ranaivoson, Gary W. Feyereisen, Carl J. Rosen, John F. Moncrief

**Affiliations:** 1 Department of Soil, Water, and Climate, University of Minnesota, Saint Paul, Minnesota, United States of America; 2 USDA Agricultural Research Service, Soil and Water Management Research Unit, Saint Paul, Minnesota, United States of America; University of Siena, ITALY

## Abstract

Transport of nitrogen and phosphorus from agricultural and urban landscapes to surface water bodies can cause adverse environmental impacts. The main objective of this long-term study was to quantify and compare contaminant transport in agricultural drainage water and urban stormwater runoff. We measured flow rate and contaminant concentration in stormwater runoff from Willmar, Minnesota, USA, and in drainage water from subsurface-drained fields with surface inlets, namely, Unfertilized and Fertilized Fields. Commercial fertilizer and turkey litter manure were applied to the Fertilized Field based on agronomic requirements. Results showed that the City Stormwater transported significantly higher loads per unit area of ammonium, total suspended solids (TSS), and total phosphorus (TP) than the Fertilized Field, but nitrate load was significantly lower. Nitrate load transport in drainage water from the Unfertilized Field was 58% of that from the Fertilized Field. Linear regression analysis indicated that a 1% increase in flow depth resulted in a 1.05% increase of TSS load from the City Stormwater, a 1.07% increase in nitrate load from the Fertilized Field, and a 1.11% increase in TP load from the Fertilized Field. This indicates an increase in concentration with a rise in flow depth, revealing that concentration variation was a significant factor influencing the dynamics of load transport. Further regression analysis showed the importance of targeting high flows to reduce contaminant transport. In conclusion, for watersheds similar to this one, management practices should be directed to load reduction of ammonium and TSS from urban areas, and nitrate from cropland while TP should be a target for both.

## 1. Introduction

Nitrogen (N) and phosphorus (P) are essential for plant and animal growth. However, when elevated concentrations of these nutrients get into surface water bodies, they can cause adverse environmental impacts such as hypoxia and harmful algal blooms[1–3]. Consequently, these outcomes cause societal, ecological, and economic concerns. Therefore, it is important to understand the extent of nutrient pollution transport from various sources, so best management practices can be effectively targeted at their source.

Commercial fertilizer and manure have been identified as important sources of nutrients to surface water degradation in the USA with the highest application rates occurring over a wide region of the Upper Midwest[4]. Farmers in humid and semi-humid regions apply fertilizers for profitable crop production, but some of the N and P can rapidly be transported to surface water bodies via subsurface drainage[5–8]. Another possible path of nutrient transport to surface water bodies is surface inlets (sometimes called open inlets) that connect to subsurface drainage systems[9,10]. The subsurface drainage path of nutrient transport from cropland with manure and inorganic fertilizer application has been well documented in plot[11,12], and on-farm experiments[13–16].

Another non-point source of nutrient pollution in surface water bodies is stormwater runoff from urban watersheds[17–19]. Atmospheric deposition, application of fertilizers to lawns, leaf litter, grass clippings, sanitary sewer leakage due to aged infrastructure, and pet waste are sources of N and P in stormwater runoff from urban areas[20,21]. Previous studies have reported nutrient transport in stormwater runoff from urban watersheds[19,20,22].

Based on the above-mentioned literature review, non-point source pollution of agricultural drainage water and urban stormwater runoff have been well documented in separate studies around the world. However, the extent of nutrient transport from these non-point sources has rarely been investigated over the long term within the same watershed. If informed management decisions for nutrient reduction strategies in a watershed are to be done effectively, a better understanding of the extent of nutrient transport from agricultural and urban landscapes to the receiving surface water body will be needed.

Harmful algal blooms are a concern in fresh water bodies due to the potential for toxin production, which is a serious concern to public health and other aquatic organisms[3,23]. In Minnesota, USA, the same concern associated with harmful algal blooms in Lake Wakanda located near Willmar has been reported[24]. Although commercial fertilizer and manure are important sources of nutrient pollution that can cause harmful algal blooms, the extent of water quality degradation due to fertilizer application compared to the case without fertilizer is not well documented in subsurface-drained soils. Comparison of nutrient transport from a fertilized field (e.g. turkey manure, anhydrous ammonia, ammoniated phosphates, etc.) with an unfertilized field will help guide nutrient management best practices at the field scale. In Minnesota, these results will be useful since this state is the largest turkey producer in the USA[25], and farmers frequently use turkey manure (i.e., usually applied in fall) as a source of N and P for crop production. Therefore, there is a need to quantify and compare nutrient transport in drainage water from fertilized and unfertilized farmland.

One challenge of analyzing time series data from various systems (e.g., subsurface drainage, watershed, etc.) is the presence of serial correlation that results in lack of the independence assumption. When serial correlation is present, the significance test and R-square values are no longer accurate[26]. Although adjustment for serial correlation is important, it is not routinely done in studies reported in the literature. Therefore, we used robust statistical methods to adjust for serial correlation and accomplish our goals.

The objectives of this observational study were to conduct a long-term experiment to (1) compare and quantify contaminant transport from a fertilized farmland to an urban landscape in the same watershed, (2) determine the extent of contaminant transport from an unfertilized field and a fertilized field, and (3) assess the dynamics of contaminant load transport from fertilized fields, unfertilized fields and an urban landscape.

## 2. Materials and Methods

### 2.1. Site description

**Fig 1:**
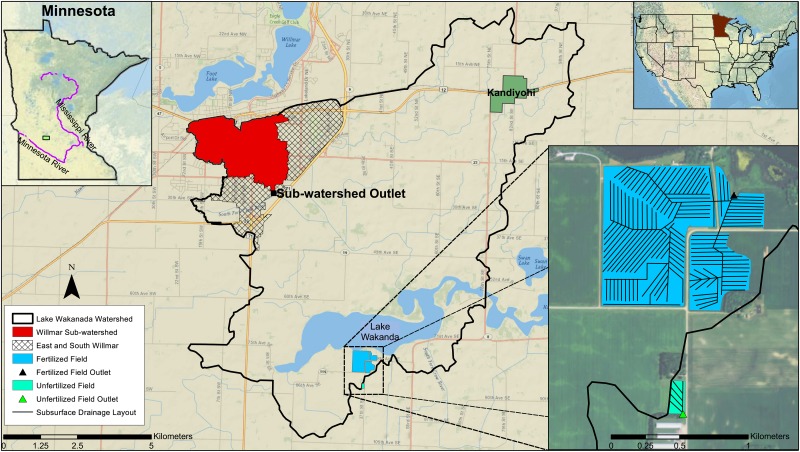
Location of the Willmar sub-watershed, Fertilized and Unfertilized Fields in Minnesota, USA.

We conducted this research on a private farm near Willmar, Minnesota, USA, from 2007 to 2013 (Fig 1). Permission was granted by the landowner to conduct the research on his farm. The farm consisted of two fields, including an Unfertilized Field (UF) (45° 02' 24" N and 95° 59' 33" W) with a contributing area of 1.3 ha and a Fertilized Field (FF) (45° 03' 01" N and 95° 59' 21" W) with 50.0 ha area. The Unfertilized Field area was small to limit economic losses to the farmer. The dominant soil type in the fields is Canisteo-Harps loam, which is a poorly drained soil[27]. The taxonomic class for Canisteo is fine-loamy, mixed, calcareous, mesic Typic Endoaquolls, and for Harps is fine-loamy, mixed, mesic Typic Calciaquolls[28]. The selected soil chemical properties are summarized in Table 1.

**Table 1:** Soil chemical properties of the Fertilized and Unfertilized Fields collected by crop consultants.

Description	Soil depth (cm)	Fertilized Field				Unfertilized Field	
		5 Oct 2007 (n = 2)	22 Oct 2008 (n = 20)^a^	31 Oct 2011 (n = 7)	10 Oct 2012 (n = 7)	22 Oct 2008 (n = 4)^a^	31 Oct 2011 (n = 1)
Soil nitrate-N (mg kg^-1^)	0–60	4.3	5.7	3.9	5.3	1.8	2.0
Soil P Olsen (mg kg^-1^)	0–15	13.5	19.3	18.8	9.1^b^	15	15
Soil pH	0–15	7.8	7.9	8.0	8.0	7.9	7.8
Soil organic matter, %	0–15	4.6	5.1	5.7	4.8	5.2	4.2

^a^ Based on grid that represents the entire field.

^b^ The low soil P is likely due to the low P fertilizer rate applied in fall 2011 and crop P uptake in the 2012 growing season.

Each field was subsurface drained with perforated corrugated plastic drain pipes (100 mm diameter) installed at an average 1.2 m depth with 24 m spacing. In addition, the Unfertilized and Fertilized Fields had four and one surface inlets, respectively, that connected to the subsurface drainage system. The slope near the surface inlet for the Unfertilized Field was 0.0073 m m^-1^, and ranged from 0.0041 to 0.0076 m m^-1^ for the Fertilized Field. Drainage water from the Fertilized Field flowed into a sump from which it was pumped into a wetland before entering Lake Wakanda. Drainage water from the Unfertilized Field flowed into a sump from which it was pumped into a sub-main pipe that conveyed the water away from Lake Wakanda.

Stormwater runoff (referred to as City Stormwater) from a sub-watershed with an area of 568 ha, that comprises central Willmar, Minnesota, USA (45° 05' 57" N and 95° 02' 00" W), was compared with the Fertilized Field (Fig 1). Permission was granted by Kandiyohi Drainage Authority to conduct the research at the City Stormwater site. The sub-watershed area was calculated based on a storm sewer map[29] and 2010 LiDAR data (i.e., 1-meter digital elevation model)[30] using ArcMap version 10.3 (ESRI Inc., Redlands, California). We calculated the impervious surfaces for the urban sub-watersheds using ArcMap based on the National Land Cover Database 2011[31]. Land use in the Willmar sub-watershed was approximately 30% residential, 38% business and governmental, and 2% parks[32].

Flow from the Fertilized Field and City Stormwater eventually enters Lake Wakanda. The Lake Wakanda watershed has a total area of 10,144 ha (including the lake) of which 1,290 ha is urban and 5,963 ha is cultivated cropland. The urban areas were comprised of east and south Willmar (622 ha with 39% impervious surface) and Kandiyohi (101 ha with 30% impervious surface) based on the city limits[33] as well as the Willmar sub-watershed (568 ha with 54% impervious surface). The outflow from the Willmar Wastewater Treatment Facility does not flow into the Lake Wakanda watershed. The state of Minnesota restricts the application of phosphorus fertilizer to residential lawns[34].

### 2.2. Precipitation measurement

On-site daily precipitation measurements were recorded at the Fertilized Field and City Stormwater sites using tipping bucket rain gauges (model TR-5251, Texas Electronics Inc., Dallas, Texas, USA). Missing daily precipitation data were replaced with that from the closest weather station located in the city of Willmar, Minnesota[35]. The years from 2007 to 2013 encompassed a range of climatic conditions, which included dry and wet years relative to the long-term normal precipitation (Table 2).

**Table 2:** Precipitation from 1 April to 31 October for the City Stormwater and Fertilized Field.

Site	Precipitation (mm)								
	2007	2008	2009	2010	2011	2012	2013	Average 2007–13	30-year normal^a^
City Stormwater	628	578	584	766	662	429	593	606	619
Fertilized Field	639	592	611	837	592	466	580	617	

^a^ Long-term normal is from 1981 to 2010 (National Oceanic and Atmospheric Administration).

### 2.3. Crop, fertilization management, and tillage

For the Fertilized and Unfertilized Fields, the crop planting sequence started with soybean in 2007 (*Glycine max* L.) followed by two years of corn (*Zea mays* L.). Soybean was planted again in 2010 to complete two crop rotation cycles (Table 3). Commercial fertilizer and turkey litter manure were applied to the Fertilized Field. Turkey manure application rates were based on the manure N content, crop N needs and soil test. The commercial fertilizer application rates were based on the crop N needs and soil test. Neither fertilizer nor manure had been applied to the Unfertilized Field since fall of 2005.

**Table 3:** Summary of crop sequence, fertilizer type and application rate, and tillage system for the Fertilized Field.

Description	Crop						
	Soybean	Corn	Corn	Soybean	Corn	Corn	Corn
Fertilizer type	None	Turkey litter & commercial fertilizer^e^	Commercial fertilizer	None	Turkey litter & commercial fertilizer^e^	Commercial fertilizer	Commercial fertilizer
Fertilizer application date	None	11 Oct 2007	11 Nov 2008	None	1 Oct 2010	4 Nov 2011	18 Oct 2012
Nitrogen application rate (kg ha^-1^)	None	199	144^a^^,^^b^	None	177^f^	170^b^^,^^d^	147^b^^,^^d^
Phosphorus application rate (kg ha^-1^)	None	57	8^b^	None	81	4^b^	12^b^
Potassium application rate (kg ha^-1^)	None	82	61^c^	None	111	43^c^	20
Tillage	Fall 2006 chisel plow	Fall 2007 moldboard plow	Fall 2008 chisel plow	Fall 2009 chisel plow	Fall 2010 moldboard plow	Fall 2011 chisel plow	Fall 2012 moldboard plow
Planting date	16 May 2007	5 May 2008	2 May 2009	6 May 2010	18 May 2011	27 Apr 2012	30 Apr 2013
Harvest date	4 Oct 2007	18 Oct 2008	17 Nov 2009	14 Sep 2010	28 Oct 2011	6 Oct 2012	25 Sep 2013

^a^ Anhydrous ammonia.

^b^ Diammonium phosphate.

^c^ Potash.

^d^ Urea.

^e^ 61% and 39% of nitrogen was from manure and anhydrous ammonia, respectively. P and K were from manure.

^f^ 55% and 45% of nitrogen was from manure and anhydrous ammonia, respectively. P and K were from manure.

Tillage practices varied depending on the crop. For the Fertilized Field, following soybean, turkey manure was incorporated with a chisel plow (i.e., parabolic shanks following discs to chop stalks). After first-year corn, a moldboard plow was used in the fall to incorporate fertilizer within the top 0.3 m of the soil. After second-year corn, fields were fall chisel plowed. Since the field was left rough after fall chisel and moldboard plowing, it was tilled with a field cultivator in the spring to break up soil clumps and provide a seedbed. For the Unfertilized Field, fall chisel plow was used after corn harvest, and the field cultivator was used in spring before planting corn and soybean. For both fields, corn and soybean were planted at a planting density of 82,750 and 329,750 seeds ha^-1^, respectively, at a row spacing of 0.56 m.

### 2.4. Contaminant and flow monitoring

We monitored nitrate (NO_3_^-^), ammonium (NH_4_^+^), total suspended solids (TSS), and total phosphorus (TP) along with flow rate at all three sites. Each site had an automated data acquisition system comprised of a data logger (models 21X and CR10, Campbell Scientific Inc., Logan, Utah, USA) to collect water temperature and flow rate readings. Each site had an automated sampler (6700 series, Teledyne ISCO, Lincoln, Nebraska, USA) to collect up to twelve pairs of bottles, one bottle with acid and the other acid-free. Each bottle was comprised of twelve 80 mL sub-samples, and each pair was collected over a sampling time. We programmed the automated samplers to have a minimum of 2 days per bottle pair at the beginning of the study, and a minimum of 4 days per bottle pair midway through the study. This procedure provided us with daily contaminant concentrations, which were used to calculate daily contaminant loads. For the field sites, the sampling period varied with flow rate (i.e., flow proportional sampling). For the City Stormwater site, the sampling period was time based. The automated samplers were located inside refrigerators (4°C), from which samples were retrieved weekly.

For the Unfertilized Field, flow rate was measured in a pipe every minute using a paddlewheel flow sensor (model FP-5300, Omega, Stanford, Connecticut, USA). For the Fertilized Field, we measured flow rate in a pipe every minute using two Area Velocity Flow Loggers with ISCO model 4150 (used earlier in study) and ISCO 2150 (used later in the study) (Teledyne Isco Inc., Lincoln, Nebraska, USA). For the City Stormwater, we used Area Velocity Flow Loggers model 4150 and/or 2150 (Teledyne Isco Inc., Lincoln, Nebraska, USA) to measure water velocity and height inside a rectangular concrete culvert (9.4 m wide, 3.0 m high, and 32 m long) located at the outlet of the Willmar sub-watershed in a drainage ditch (S1 Multimedia). The water height was used to calculate the cross sectional area, which was multiplied by water velocity to yield flow rate.

For the field sites, no data were collected during the time the ground was frozen (i.e., sometime between November to March), since there was no drainage flow during this period as the water in both sumps was frozen. For the City Stormwater, freezing conditions prevented flow rate measurement. At all sites, we started collecting data shortly after ice/snow thaw in late March as feasible. Consequently, we used data from 1 April through 31 October of 2007 to 2013 for all sites, except for the City Stormwater where 2008 and 2013 data were not included in any analyses due to equipment failure. For the City Stormwater, it may have been possible that we missed data during short periods of ice/snow thaw before sensors were put in place, though these data are expected to be negligible relative to the vast majority of flow that occurred after 1 April.

### 2.5. Water sample analysis

Water samples were analyzed for nitrate according to the automated cadmium reduction method in section 4500-NO_3_^-^, and ammonia according to the ammonia-selective electrode method in section 4500-NH_3_ of Rice et al.[36] Since ionized ammonia (i.e., ammonium, NH_4_^+^) is the predominant form in stormwater and field runoff, we will use the term ammonium[37,38]. TSS was analyzed based on USGS I-3765-85, and TP based on USEPA Method 365.3 (Persulfate Digestion). All water samples were analyzed at the Stearns DHIA Laboratories in Sauk Centre, Minnesota. The acid-preserved samples were used to analyze for nitrate, ammonium and TP, and the acid-free samples for TSS. When the concentrations of ammonium and TSS were below detection limit, we used half the detection limit as their concentration. The detection limit for ammonium was 0.100 mg L^-1^ from 2007 to 2008, 0.045 from 2009 to 2011, 0.072 for 2012, 0.069 for 2013, and the detection limit for TSS was 1.0 mg L^-1^. The concentrations of nitrate and TP were always above their detection limits of 0.016 and 0.005 mg L^-1^, respectively.

### 2.6. Data analysis

#### 2.6.1. Contaminant concentration trend

We used the seasonal Mann-Kendall (non-parametric) trend test to determine whether there was a trend in the time series data of daily contaminant concentrations for the Unfertilized and Fertilized Fields over the period of the study. This test takes into account the seasonality of the data and accounts for autocorrelation or serial correlation. If a general monotonous trend (i.e., increasing or decreasing) is significant for a given time series, the Mann-Kendall quantifies the slope (also known as “Sen Slope”) of the trend. We performed the seasonal Mann-Kendall trend using XLSTAT (Addinsoft SARL, New York, NY). A 5% confidence level was used to determine statistical significance. For the City Stormwater, the sample size was not large enough to conduct a general trend test due to missing years.

#### 2.6.2. Precipitation, flow, and contaminant load comparison

For all sites, we normalized flow rate (section 2.4.) by dividing the daily flow volume by the drainage area to yield daily flow depth, which eliminates the effect of differences in drainage area. We calculated daily contaminant load per area by dividing the product of contaminant concentration and daily flow volume by the drainage area. We used a paired *t*-test to compare daily contaminant loads and daily flow depths between the City Stormwater and Fertilized Field, and between the Unfertilized and Fertilized Fields. Furthermore, we used the same procedure to compare daily precipitation between the City Stormwater and Fertilized Field. In these comparisons, we used daily values whether or not they were zero. In this period, when daily data were missing from one site due to equipment failure, those days were not used in the paired comparisons (S1 File).

The normality assumption required for the paired *t*-test was met based on the Central Limit Theorem due to the large sample size[39]. The independence assumption of the error terms was not satisfied, since the Durbin-Watson statistic was different from two (S2 File). In other words, the error terms were not independent over time due to serial correlation. To account for this effect, we adjusted the standard error equation as[39]

$$SE=\sqrt{\frac{1+r_{1}}{1-r_{1}}}\frac{s}{\sqrt{n}}$$ (1)

where *s* is the standard deviation of the paired differences, n is the number of days (sample size), and *r*_1_ is the first serial correlation coefficient. Under the condition where *r*_1_ is zero (i.e., no serial correlation), the above equation becomes the usual standard error equation. This serial correlation adjustment is valid under the first-order autoregressive model or AR(1). Therefore, the adequacy of the AR(1) model was confirmed by observing an abrupt cut off of the partial autocorrelation function after lag 1.

The first serial correlation coefficient (*r*_1_) is calculated as the ratio of *c*_1_ to *c*_0_, which are calculated as

$$c_{1}=\frac{1}{n-1}\sum_{t=2}^{n}\left(res_{t}\times res_{t-1}\right)$$ (2)

$$c_{0}=\frac{1}{n-1}\sum_{t=1}^{n}res_{t}{}^{2}$$ (3)

where *res*_*t*_ is the residual (error estimate) at time *t*. To our knowledge, this is the first time this robust method has been used to account for serial correlation in subsurface drainage data.

Furthermore, we inspected the stationary assumption of the time series data using the augmented Dickey-Fuller unit root test. In addition, the stationary assumption was met by observing no trend (i.e., a strong and slowly dying trend was not observed) in the autocorrelation function[26]. We performed the paired *t*-test and the assumption checks using JMP Pro 12 (SAS Institute Inc., Cary, NC).

#### 2.6.3. Contaminant transport dynamics

To assess the contaminant transport dynamics, we evaluated the relationship between contaminant load and flow depth at each site over the period of the study. This analysis also allows for comparison of the water quality implications among sites. In this analysis, we fitted a linear regression to the plot of the natural logarithm of daily contaminant load, *L* (kg ha^-1^) versus natural logarithm of daily flow depth, *D* (mm) which is written as

Ln L=b Ln D+Ln a (4)

where the slope *b* is the elasticity coefficient, and *a* is a constant. When the slope is one, the nutrient concentration remains constant with varying flow depth. When the slope is greater than one, high flows lead to increased concentration, and/or low flows lead to lower concentrations[40,41]. Moreover, the slope is the percent increase in contaminant load induced by 1% increase in flow depth.

The Durbin-Watson statistic showed that the independence assumption of the error terms for the ordinary least squares linear regression of the daily values was violated. Thus, we used average weekly values to minimize serial correlation, but a significant effect was still present according to the Durbin-Watson statistic. When serial correlation is present, the significance test and R-square values are no longer accurate[26]. Although the regression coefficients of an ordinary least squares estimate are unbiased, they are not minimum variance estimates when serial correlation is present. Consequently, we used the AUTOREG procedure of SAS 9.4 (SAS Institute Inc., Cary, NC) with the option of METHOD = ML (i.e., exact maximum likelihood method) for daily values to estimate the parameters of the linear regression[26]. The NLAG = *number* option was also used to specify the order of the autoregressive model. The order of the autoregressive model was either one or two for all the regression analyses.

We validated the stationarity assumption using the augmented Dickey-Fuller unit root test. The normality assumption of the linear regression was met based on the Central Limit Theorem[39]. The equal variance assumption was confirmed by observing no structure in the plot of the residuals versus fitted values of the autoregressive model. We performed the regression analyses and assumption checks using SAS 9.4 (SAS Institute Inc., Cary, NC).

To determine whether the slopes were significantly different from one, we constructed the 95% confidence interval. If the confidence interval did not include one, the difference from one was statistically significant. We also used the 95% confidence intervals to compare the slopes among sites. If the confidence interval of the slopes were not overlapping, their difference was statistically significant.

## 3. Results and Discussion

### 3.1. Precipitation, flow depth, and crop yield comparisons

#### 3.1.1. Precipitation for City Stormwater and Fertilized Field

Based on the days where both sites had flow data from April to October, the average (± SD) daily precipitation values for the City Stormwater and Fertilized Field were 3.0 ± 9.0 and 3.1 ± 9.5 mm, respectively. Although the sites were approximately 8 km apart, their average daily precipitations were not significantly different based on the adjusted paired *t*-test (two-sided *p*-value = 0.702, n = 792). Therefore, we conclude that any difference in flow depth between the two sites is not due to precipitation.

#### 3.1.2. Flow for City Stormwater and Fertilized Field sites

**Fig 2:**
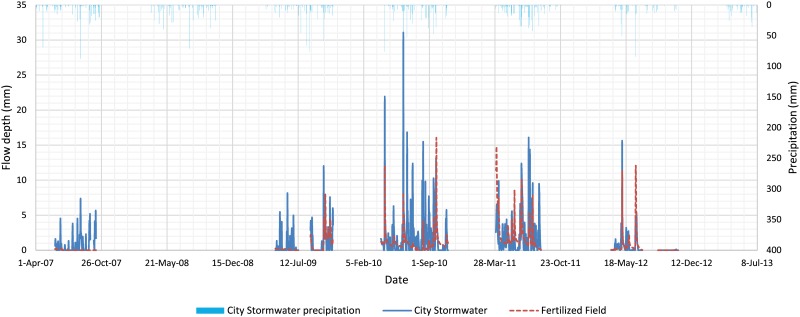
Daily flow depth for City Stormwater and Fertilized Field (excluding 2008 and 2013) used for the paired *t*-test, and daily precipitation at the City Stormwater.

The total flow depths for the City Stormwater and Fertilized Field were 1110 and 753 mm, respectively (Fig 2). These flow depths represent 46.0% (1110/2411) and 30.5% (753/2467) runoff of the total precipitation over this period, respectively. Table 4 summarizes the results from the paired *t*-test comparing flow depth between the City Stormwater and Fertilized Field after adjusting for serial correlation. Results showed that the average daily flow depth for the City Stormwater was significantly higher than that for the Fertilized Field, which was expected due to the 54% impervious area in the Willmar sub-watershed.

**Table 4:** Cumulative flow depth from April to October of 2007 to 2012 (excluding 2008), and paired *t*-test comparison between Stormwater and Fertilized Field after adjusting for serial correlation (n = 792).

Description	Cumulative flow depth (mm)	Average daily flow depth ± SD (mm)	First serial correlation coefficient, r_1_	One-sided *p*-value
City Stormwater	1110	1.40 ± 2.69	0.335	<0.001
Fertilized Field	753	0.95 ± 1.80		

#### 3.1.3. Flow for Unfertilized and Fertilized Fields

**Fig 3:**
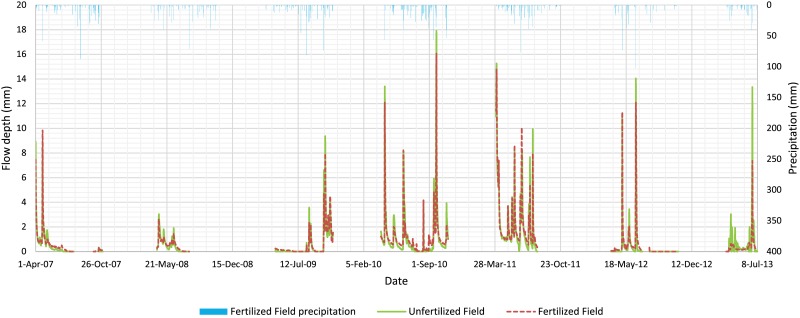
Daily flow depth for Unfertilized and Fertilized Fields used for the paired *t*-test, and daily precipitation at the Fertilized Field.

Average daily flow depths from April to October of 2007 to 2013 for the Unfertilized and Fertilized Fields were 0.86 ± 1.83 and 0.87 ± 1.65 mm (918 and 932 mm cumulative), respectively, which were not significantly different based on the paired *t*-test, adjusted for serial correlation (two-sided *p*-value = 0.621, n = 1071). Furthermore, Fig 3 shows that the two drainage systems had similar daily flow responses to the same daily precipitation.

The fact that these two sites had similar drainage flow responses (Fig 3) and that average daily flow depths were not significantly different indicates that the drainage intensity of the two drainage systems were similar. The drainage intensity is calculated by the Hooghoudt equation and is dependent on the effective saturated hydraulic conductivity of the soil profile, drain depth, drain spacing, effective radius of the drain, and drain depth to restrictive soil layer[42]. In other words, the drainage systems of the two fields performed similarly. Therefore, differences in load transport from the two fields cannot be induced by the drainage systems. However, differences in the ratio of closed-depression area (drained by surface inlets) to the entire field may affect sediment runoff. Overall, the results of this section are essential for our observational study because it establishes a cause-and-effect relationship between nutrient transport and fertilizer application.

#### 3.1.4. Crop yield for Unfertilized and Fertilized Fields

Corn and soybean yields were calculated each year by averaging the unfiltered (i.e., outliers were not removed) yield monitor data[43]. The five-year average corn yields for the Fertilized and Unfertilized Fields were 11.38 Mg ha^-1^ and 7.02 Mg ha^-1^, respectively (S3 File). The two-year average soybean yields for the Fertilized and Unfertilized Fields were 3.43 and 3.83 Mg ha^-1^, respectively. As expected, corn yield was lower in the Unfertilized Field compared with the Fertilized Field, which is most likely due to lack of nitrogen from withholding of fertilizer and manure. However, we did not observe such an effect for soybean because this plant is a legume that fixes atmospheric nitrogen.

### 3.2. Contaminant concentration in drainage water and stormwater runoff

#### 3.2.1. Nitrate concentration

**Fig 4:**
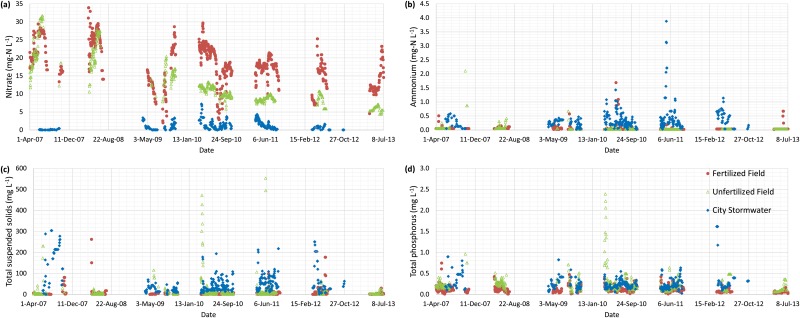
Daily contaminant concentrations from 2007 to 2013 for the City Stormwater (excluding 2008 and 2013), Unfertilized and Fertilized Fields for (a) nitrate, (b) ammonium, (c) total suspended solids, and (d) total phosphorus.

**Fig 5:**
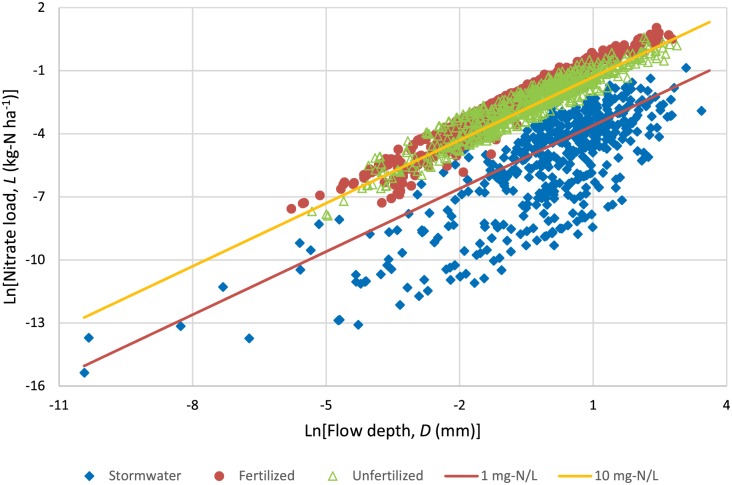
Relationship between daily nitrate load and daily flow depth over the period of the study for City Stormwater (n = 479), Fertilized Field (n = 907), and Unfertilized Field (n = 663). The lines represent the loads at constant nitrate concentration of 1 and 10 mg-N L^-1^.

Fig 4 presents the daily contaminant concentration over the period of the study. The median nitrate concentration for the Fertilized Field (17.88 mg-N L^-1^) was 1.7 times higher than that of the Unfertilized Field (10.49 mg-N L^-1^). This difference is also apparent from Fig 5, which is discussed in section 3.5.1. The higher median nitrate concentration for the Fertilized Field than the Unfertilized Field was most likely caused by the application of manure and commercial fertilizer. The impact of fertilizer application on the soil can be seen in the higher soil nitrate-N test in the Fertilized Field than the Unfertilized Field in fall 2008 (i.e., based on grid sampling) (Table 1). The source of nitrate from the Unfertilized Field could be attributed to legacy nitrate from previous fertilizers and mineralization of soil organic matter as this has been reported to be a substantial source[44]. Nguyen et al.[45] also reported higher 12-year average flow-weighted nitrate concentration for fertilized plots than an unfertilized plot, although the extent of the concentration difference due to fertilizer application cannot be determined because their flow depths were significantly different between plots. Thus, their flow-weighted nitrate concentration was affected by both flow depth and application of fertilizer.

The City Stormwater nitrate concentration ranged from 0.01 to 7.22 mg-N L^-1^ with a median of 0.64 mg-N L^-1^, which was close to the range of 0.05 to 6.7 mg-N L^-1^ from a review of highway runoff from various locations worldwide[46]. The median nitrate concentration in our study was also similar to that of 0.60 mg-N L^-1^ from 360 stormwater sites throughout the USA[47].

The median nitrate concentration for the Fertilized Field was 27.9 times (17.88/0.64) higher than that for the City Stormwater, which can be explained by the greater inorganic and organic sources of N in the soil. This difference in nitrate concentration is also evident from Fig 5, which is discussed in section 3.5.1. With respect to the lower nitrate concentration for the City Stormwater, Kaushal et al.[48] reported decline in nitrate concentrations with rise in urbanization. Fissore et al.[21] found fertilizer application to lawns as the major source of N followed by atmospheric deposition and pet waste in an urban watershed in Minnesota, USA.

It is important to note that this research is an observational study. In these studies, although statistical methods cannot eliminate the possibility of confounding factors, there may be strong theoretical reasons for establishing a causation in other ways[39]. For example, the reason for the higher nitrate concentration in the Fertilized Field than the Unfertilized Field was most likely due to the application of manure and commercial fertilizer, since other factors were similar (section 3.1.3.).

#### 3.2.2. Ammonium concentration

Ammonium concentration was below detection limit for 86.1% (571/663) and 83.7% (759/907) of the total number of days for Unfertilized (up to 2.11 mg-N L^-1^) and Fertilized Fields (up to 1.69 mg-N L^-1^), respectively (Fig 4). Thus, the median concentration for each site (0.03 mg-N L^-1^) was very low. Since the soil has a moderate pH and temperature condition, the vast majority of the total ammonia will be in the ammonium form (ionized ammonia)[37]. As a result, ammonium is less prone to transport via subsurface drainage, since it is bound to soil particles due to its positive charge. Our result was close to the range of below detection limit to 0.99 mg-N L^-1^ reported by Hofmann et al.[49], which was measured in fertilized subsurface-drained plots in Indiana, USA. Ammonia generated from livestock operations is usually deposited in local areas[50].

The median ammonium concentration for the City Stormwater was 0.27 mg-N L^-1^ with a range of 0.02 to 3.88 mg-N L^-1^, which was 9.0 times (0.27/0.03) higher than that for the Fertilized Field (Fig 4). Our result is close to the range of 0.27 to 4.60 mg-N L^-1^ for concentration in highway runoff from various locations worldwide[46]. Others have also reported similar median ammonium concentrations to our study. Passeport and Hunt[18] reported median ammonia concentration of 0.22 mg-N L^-1^ from highway runoff. The median ammonium concentration from 360 stormwater sites throughout the USA was 0.44 mg-N L^-1^ [47].

One possible source of the higher ammonium concentration for the City Stormwater is atmospheric deposition of ammonia from catalytic converters in automobiles under high fuel to air ratios during acceleration[51]. Maestre and Pitt[47] showed that ammonia concentration is higher on highways among different land uses in the USA, suggesting a relationship between ammonia concentration and vehicles. Furthermore, Heeb et al.[52] provided evidence of correlation between ammonia concentration and acceleration. Therefore, one source of the ammonium for the City Stormwater is likely localized ammonia deposition from automobiles, which takes the form of ammonium in stormwater under moderate pH and temperature[37]. Other important sources of ammonium are from rainfall[22] and atmospheric deposition[53]. Zhang et al.[53] reported that anthropogenic sources accounted for the majority of the NHx deposition to the contiguous USA.

The ammonium from the City Stormwater flows into Lake Wakanda, which can become toxic to aquatic life (e.g., fish), if pH and water temperature of the lake increases. Duration and frequency of exposure of fish to ammonia are also important factors affecting fish toxicity[54]. Further information regarding ammonium concentration in Lake Wakanda is needed to assess if the concentrations are at harmful levels to fish and other aquatic life, but it is certain that urban stormwater is a source of ammonium.

#### 3.2.3. Total suspended solids concentration

The median TSS concentration for the Unfertilized Field (5.0 mg L^-1^) was 2.2 times higher than that for the Fertilized Field (2.3 mg L^-1^) (Fig 4). This can be explained by the observation of lesser crop biomass, and therefore, smaller crop residue in the Unfertilized Field that did not provide sufficient plant residues for erosion protection (S1 and S2 Figs). Another potential factor that could have contributed to differences in TSS is the ratio of closed-depression area (drained by surface inlets) to the entire field.

The median TSS concentration for the City Stormwater was 35.0 mg L^-1^ with a range of 0.5 to 304.0 mg L^-1^, which was 15.2 times (35.0/2.3) higher than that of the Fertilized Field (Fig 4). This is because the impervious area of the city (i.e., 54% of city area) provides more opportunity for the sediments to be transported in runoff. The TSS concentration in highway runoff from various locations worldwide ranged from 46 to 476.3 mg L^-1^ [46]. The median TSS concentration from 360 stormwater sites throughout the USA was 59 mg L^-1^ [47].

#### 3.2.4. Total phosphorus concentration

The median TP concentration of 0.19 mg L^-1^ (range of 0.04 to 2.39 mg L^-1^) for the Unfertilized Field was 1.9 times higher than the median of 0.10 mg L^-1^ for the Fertilized Field (range 0.01 to 0.75 mg L^-1^) (Fig 4). Our TP concentrations were slightly lower than those reported by Madison et al.[55] with flow weighted TP concentrations ranging from 0.55 to 1.76 mg L^-1^ from two subsurface-drained farms under chisel-plowed continuous corn silage and dairy manure application in Wisconsin, USA. Since the soil P test was lower in the Unfertilized Field than the Fertilized Field in fall 2008 (i.e., based on grid sampling) (Table 1) and the fact that TP includes particulate P, we speculate that the higher TP concentrations in the drainage water of the Unfertilized Field are associated with its higher TSS. In other words, the higher TSS of the Unfertilized Field may have transported higher concentrations of TP (in the form of particulate P) to the surface inlets[56].

The City Stormwater had a median TP concentration of 0.22 mg L^-1^ (range of 0.02 to 1.62 mg L^-1^) that was 2.2 times higher than that of the Fertilized Field (Fig 4). The median TP concentration for the City Stormwater was very close to values reported in other locations. In a highway runoff review from various locations worldwide, TP concentration ranged from 0.13 to 0.91 mg L^-1^ [46]. The median TP concentration from 360 stormwater sites throughout the USA was 0.27 mg L^-1^ [47].

Phosphorus is transported in particulate and dissolved forms. Though the particulate P is not bioavailable, it is important since desorption of P from sediments and soil can occur under the right conditions[20], and in turn, cause eutrophication of surface water. Based on a review of highway runoff by Kayhanian et al.[46], particulate P has been found to be the dominating form of P. Therefore, the higher TP concentration for the City Stormwater may be related to its higher TSS concentration, which may have transported mostly particulate P with sediments. This is because impervious areas in urban watersheds provide more opportunity for sediments to be transported in stormwater runoff[57]. In other words, the lack of P retention on impervious surfaces combined with soil erosion from the ground, ditches (i.e., about 1.95 km length), and construction sites could be a cause of the high TP concentration for the City Stormwater. Duan et al.[20] also attributed high TP concentration to erosion caused by stormwater. Another reason for the high TP concentration in our study could be sanitary sewer leakage, which was found to be an important source of stormwater contamination in Maryland, USA[20].

### 3.3. Concentration trend comparisons

For nitrate, we found a significant general negative trend (two-sided *p*-value<0.001) for the Fertilized and Unfertilized Fields in the drainage outflow, which means that nitrate concentration has significantly decreased over seven years (Fig 4a). The reason for the decreased nitrate concentration may be due to improved nutrient management practices implemented by the farmer. Though the slope of the Unfertilized Field (-0.022 mg-N L^-1^ day^-1^) was steeper than that of the Fertilized Field (-0.013 mg-N L^-1^ day^-1^), their difference was not statistically significant. The slightly steeper slope of the Unfertilized Field can be explained by the lack of fertilizer application that caused the nitrate concentration to decline more quickly.

In terms of TP, the Mann-Kendall test showed a significant negative trend (two-sided *p*-value<0.001) for the Fertilized and Unfertilized Fields. Though the slope of the Unfertilized Field (-0.00017 mg L^-1^ day^-1^) was slightly steeper than that of the Fertilized Field (-0.00006 mg L^-1^ day^-1^), their difference was not statistically significant. The fact that the study duration was long enough to evaluate trends, shows the importance of long-term research.

### 3.4. Contaminant load comparisons

#### 3.4.1. Loads from City Stormwater and Fertilized Field

*Nitrate load*. Average daily nitrate load per unit area from the Fertilized Field was significantly higher than that from the City Stormwater (Table 5). The fact that flow depth was significantly lower for the Fertilized Field than the Stormwater site (Table 4) indicates that the difference in nitrate load was the result of higher nitrate concentration for the Fertilized Field (section 3.2.1.). Average daily nitrate load per unit area from the Fertilized Field was 12.4 times (0.173/0.014) more than that from the City Stormwater during the period of the experiment. The average daily nitrate load of 0.173 kg-N ha^-1^ in this study was lower than but comparable to the daily load of 0.31 kg-N ha^-1^ from subsurface-drained and fertilized plots in Indiana, USA[49].

**Table 5:** Cumulative load from April to October of 2007 to 2012 (excluding 2008), and paired *t*-test comparisons of daily loads between City Stormwater and Fertilized Field after adjusting for serial correlation (n = 792).

Description	Cumulative load (kg ha^-1^)	Average daily load ± SD (kg ha^-1^)	First serial correlation coefficient, r_1_	One-sided *p*-value
Nitrate-N				
City Stormwater	11.0	0.014 ± 0.033	0.704	<0.001
Fertilized Field	136.6	0.173 ± 0.302		
Ammonium-N				
City Stormwater	3.62	0.0046 ± 0.0111	0.579	<0.001
Fertilized Field	0.34	0.0004 ± 0.0022		
Total suspended solids				
City Stormwater	768.0	0.970 ± 2.632	0.304	<0.001
Fertilized Field	64.7	0.082 ± 0.344		
Total phosphorus				
City Stormwater	2.67	0.003 ± 0.007	0.379	<0.001
Fertilized Field	1.22	0.002 ± 0.004		

*Ammonium*, *TSS*, *and TP loads*. The ammonium, TSS, and TP average daily loads per unit area from the City Stormwater were significantly higher than that from the Fertilized Field (Table 5). The higher loads were the result of higher concentrations (section 3.2.) and higher flow depth (section 3.1.2.) for the City Stormwater. These results show that urban areas are capable of transporting higher ammonium, TSS, and TP loads than farmland on a per area basis.

#### 3.4.2. Load estimates from cropland and urban areas

We made assumptions to get a rough estimate of the cumulative loads from cropland and urban areas flowing into Lake Wakanda over the study period. We assumed that, in the Lake Wakanda watershed, other agricultural fields follow the same management practices as the Fertilized Field, and other urban areas transport loads similar to the Willmar sub-watershed. Given these assumptions, cropland produced higher cumulative loads of nitrate (814,665 vs. 14,146 kg-N) and TP (7,250 vs. 3,449 kg) while the urban areas generated higher loads of ammonium (4,674 vs. 2,051 kg-N) and TSS (990,791 vs. 385,592 kg). In terms of ranking, these estimates are the same as the results of the previous section (i.e., per area basis) except for TP where cropland, with 4.6 times (5,963/1,290) higher area than urban, transported higher loads at the watershed scale.

It is important to note that these comparisons of load estimates are based on the period from April to October when vast majority of the flow occurred. Furthermore, the load estimates from cropland are not necessarily what flows into Lake Wakanda, since conservation practices such as wetlands are in place across the watershed. In the case of the Fertilized Field, drainage water enters a wetland that ultimately discharges into Lake Wakanda. The percent area of cropland in the watershed for which conservation measures exist is presently unquantified. Overall, the load estimates in this section are based on assumptions, so we advise caution in generalizing about load quantity for other watersheds.

Based on these results, similar watersheds should target reducing ammonium and TSS from urban areas, and nitrate from cropland while TP should be a focus for both. For urban areas, conventional stormwater control measures (i.e., dry and wet ponds) and more innovative measures (i.e., green roofs, permeable pavement, bioretention, vegetated open channels, sand filters and wetlands) aimed at reducing peak flow, and removing sediments and nutrients are possible solutions[58,59]. Also for cropland, controlled drainage[60], denitrification beds[61], constructed wetlands[62], and other management practices are possible solutions.

#### 3.4.3. Loads from Unfertilized and Fertilized Fields

*Nitrate load*. Average daily nitrate load per unit area from the Fertilized Field was significantly higher than that from the Unfertilized Field (Table 6). This is the result of the higher nitrate concentration for the Fertilized Field (section 3.2.1.), since flow depths were not significantly different (section 3.1.3.). Although the Unfertilized Field did not receive fertilizer nor manure since 2005, the cumulative load per area was still 58% (0.094/0.163) of that of the Fertilized Field. Nguyen et al.[45] reported nitrate load transport from subsurface-drained unfertilized and fertilized plots. However, their data do not allow quantification of the proportion of nitrate load transported due to application of fertilizer. This is because flow depth was significantly different between their plots, so the reason for the load difference between plots was both the difference in flow depth and application of fertilizer. Our results show that under management practices similar to those of this study, nitrate load in drainage water from an unfertilized field could be considerable relative to that of a fertilized field.

**Table 6:** Cumulative load from April to October of 2007 to 2013, and paired *t*-test comparisons of daily loads between Unfertilized and Fertilized Fields after adjusting for serial correlation (n = 1071).

Description	Cumulative load (kg ha^-1^)	Average daily load ± SD (kg ha^-1^)	First serial correlation coefficient, r_1_	One-sided *p*-value
Nitrate-N				
Unfertilized Field	100.6	0.094 ± 0.184	0.616	<0.001
Fertilized Field	174.2	0.163 ± 0.283		
Ammonium-N				
Unfertilized Field	0.33	0.0003 ± 0.0007	0.590	0.120^a^
Fertilized Field	0.42	0.0004 ± 0.0013		
Total suspended solids				
Unfertilized Field	210.0	0.196 ± 1.542	0.304	0.022
Fertilized Field	73.7	0.069 ± 0.306		
Total phosphorus				
Unfertilized Field	2.31	0.002 ± 0.007	0.228	0.001
Fertilized Field	1.47	0.001 ± 0.004		

^a^ Two-sided *p*-value is 0.240.

*Ammonium load*. The average daily ammonium load per unit area was not significantly different from the Unfertilized and Fertilized Fields (Table 6), which can be explained by the field having similar flow depths (section 3.1.3.) and similar median ammonium concentrations (section 3.2.2.). Results also showed that the ammonium loads from both fields were very low. Similarly, Hofmann et al.[49] considered their concentrations negligible to their overall N load loss calculations from subsurface-drained and fertilized plots in Indiana, USA.

Furthermore, our study demonstrates the importance of accounting for serial correlation in time series data. For instance, not accounting for this effect would have resulted in a one-sided *p*-value of 0.010 when comparing ammonium load of the Unfertilized and Fertilized Fields while the correct *p*-value should be 0.120. Therefore, not accounting for serial correlation can underestimate the error variance, and in turn, can considerably underestimate the *p*-value.

*TSS and TP loads*. The average daily TSS and TP loads per unit area from the Unfertilized Field were significantly higher than that from the Fertilized Field (Table 6). The significantly higher TSS and TP loads from the Unfertilized Field can be explained by the higher concentrations of these contaminants (section 3.2.), since flow depths were not significantly different (section 3.1.3.). These results showed that the Unfertilized Field, though with no fertilizer inputs, was still able to release higher TP and TSS loads than the Fertilized Field under similar flow regimes.

### 3.5. Contaminant transport dynamics

#### 3.5.1. Nitrate dynamics

The plot of the natural log of daily nitrate load (*L*) versus natural log of daily flow depth (*D*) shows that the City Stormwater had a lower nitrate concentration for both low and high flows than the Fertilized and Unfertilized Fields (Fig 5). Furthermore, the nitrate concentration at the City Stormwater was always below the nitrate concentration standard of 10 mg-N L^-1^ for drinking water in the USA[63]. A closer look at this plot also reveals that nitrate concentration in the Unfertilized Field was lower than the Fertilized Field (S4 File).

We accounted for the presence of serial correlation to obtain efficient linear regression coefficients, and unbiased R-squares and significance test *p*-values. Results showed that the slope of the Fertilized Field was significantly different from one (Table 7). For the Fertilized Field, a 1% increase in flow depth resulted in a 1.07% increase in nitrate load, revealing that nitrate concentration increased with rise in flow depth (S5 File). In terms of nitrate slope comparison among sites, the slope of the Fertilized Field was significantly greater than both the Unfertilized Field and the City Stormwater based on the 95% confidence intervals. This means that the increase in nitrate concentration with rise in flow was significantly greater for the Fertilized Field than that of the Unfertilized Field and the City Stormwater.

**Table 7:** Linear regression relating natural log of load to natural log of flow depth over the period of the study for City Stormwater (n = 479), Fertilized (n = 907) and Unfertilized Fields (n = 663) after adjusting for serial correlation.

Description	Slope, *b* ± SE	Coefficient of determination, R^2^
Nitrate-N		
City Stormwater	1.00 ± 0.014	0.92
Fertilized Field	1.07 ± 0.008*	0.95
Unfertilized Field	0.99 ± 0.003	0.99
Ammonium-N		
City Stormwater	0.97 ± 0.013*	0.93
Total suspended solids		
City Stormwater	1.05 ± 0.015*	0.92
Fertilized Field	1.01 ± 0.043	0.39
Unfertilized Field	1.08 ± 0.041*	0.52
Total phosphorus		
City Stormwater	1.01 ± 0.008	0.97
Fertilized Field	1.11 ± 0.019*	0.79
Unfertilized Field	1.02 ± 0.015	0.88

* Significantly different from one at 95% confidence level.

Furthermore, the strong linear associations (i.e., R-squares) show that hydrology has a dominant role in the transport dynamics of nitrate load at all sites. Nitrate load transport via subsurface drainage has been attributed to flow rate[64]. Thus, implementation of practices such as controlled drainage[65] that reduce drainage flow, may diminish nitrate load transport from subsurface drainage.

Our seven-year nitrate slope from the Fertilized Field was marginally greater than those reported by Tomer et al.[41] with slopes (*b*) of 1.01 and 1.06 from two subsurface-drained farms during a nine-year study in Iowa, USA, where they used weekly values to reduce serial correlation effects. Other studies have reported slopes (*b* ranging from 0.99 to 1.26), R-squares and/or *p*-values for daily nitrate load in subsurface drainage water[49,66–68], but they did not account for serial correlation. The presence of serial correlation, results in inaccurate significance tests and R-square values[26].

We also investigated the linear association between nitrate concentration and flow depth by performing a linear regression (adjusted for serial correlation) of the natural log of the two parameters (S7 File). Results showed weak linear associations (R^2^<0.08) between nitrate concentration and flow depth for all three sites. These results are similar to those of previous studies for subsurface drainage water[41,49,67,68]. Although Tiemeyer et al.[66] reported a good linear relationship between daily nitrate concentration and flow depth (i.e., R-square of 0.60 and 0.42 for two consecutive years) for a subsurface-drained plot in Germany, they did not account for serial correlation. The presence of serial correlation in regression analysis can seriously underestimate the error variance, which results in an R-square value greater than it should really be[26]. For example, in our study, an ordinary least squared linear regression between nitrate concentration and flow depth for the Fertilized Field resulted in an R-square of 0.20, whereas the correct R-square was 0.08, after accounting for serial correlation.

#### 3.5.2. Ammonium dynamics

Since ammonium concentration was below the detection limit for most of the period of the study, we refrained from determining the slope for the Fertilized and Unfertilized Fields. Reports of the slope value for ammonium in subsurface drainage are scarce. In one such study, Hernandez-Ramirez et al.[67] reported a slope of 0.98 for ammonium in a six-year study of subsurface-drained plots in Indiana, USA. For the City Stormwater, slope (*b* = 0.97) was significantly different from one, revealing that ammonium concentration decreased with rise in flow depth (Table 7). This could be due to the first flush effect that transports ammonium (i.e., from atmospheric deposition on highways) with the early portion of the stormwater runoff before reaching peak flow[46]. Another reason could have been that impervious surfaces had more ammonium (section 3.2.2.) that was transported by low flows. This is because low flows may have been generated entirely or disproportionately from impervious surfaces, whereas high flows may have been generated from both impervious and pervious surfaces. To our knowledge, there are no reports of ammonium slope for urban stormwater runoff, so this remains a topic for further investigation.

#### 3.5.3. Total suspended solids dynamics

For TSS, the slope was significantly different from one for the Unfertilized Field and City Stormwater, revealing that TSS concentration increased with rise in flow depth (Table 7). The increase at the Unfertilized Field may be due to the observed smaller crop residue, which in turn, induced high flows to transport more sediments than from the Fertilized Field. Another potential factor that could have contributed is the ratio of closed-depression area (drained by surface inlets) to the entire field. Furthermore, the linear associations (i.e., R^2^<0.52) for the Unfertilized and Fertilized Fields indicates that hydrology (i.e., flow variation) played a smaller role in the dynamics of TSS load transport than the other contaminants. For the Fertilized Field, concentration and flow variation did not play a major role in the load transport dynamics, so we recommend further investigation to identify the influential causes. For the City Stormwater, the increase in TSS concentration at high flows was likely the result of greater soil erosion from the ground, ditches (i.e., about 1.95 km length), and construction sites.

#### 3.5.4. Total phosphorus dynamics

Results showed that only the Fertilized Field had a slope significantly different from one, revealing that TP concentration increased with a rise in flow depth (Table 7). At this site, a 1% increase in flow rate resulted in 1.11% increase in TP loadh. Storm events have been reported to transport larger loads of TP from subsurface-drained fields[69]. Others have also found increased concentration of TP with increasing subsurface drainage flow per event basis in Ohio[7] and Indiana, USA[56]. In terms of slope, Madison et al.[55] reported a TP slope of 0.80 and 0.92 for two subsurface-drained farms in Wisconsin, USA. To our knowledge, our study is the first to report a TP slope significantly greater than one for subsurface drainage water.

**Fig 6:**
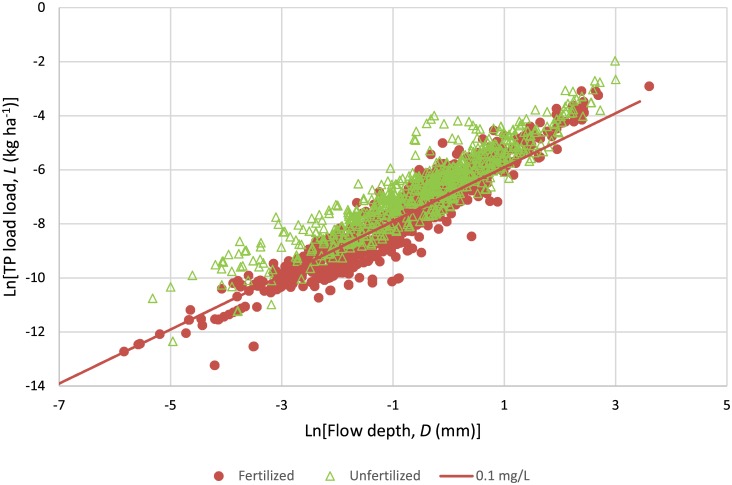
Relationship between daily total phosphorus load and daily flow depth over the period of the study for Fertilized Field (n = 966) and Unfertilized Field (n = 709). The line represents the load at constant TP concentration of 0.1 mg L^-1^.

Comparison between the Fertilized and Unfertilized Fields showed that the TP slopes of these two sites were significantly different based on the 95% confidence intervals. This difference can be observed in Fig 6, which shows that the Fertilized Field had lower TP concentrations at low flows than the Unfertilized Field, and with the increase in flow depth, both sites converge toward similar concentrations. These results show the importance of peak flows in transporting TP from subsurface-drained fields. Gall et al.[68] also noted the importance of large flow events in transporting nutrients from subsurface-drained agricultural fields.

## 4. Conclusions

The paired *t*-test, adjusted for serial correlation, showed that ammonium, TSS, and TP loads per unit area from the City Stormwater were significantly higher than that from the Fertilized Field under similar precipitation. However, nitrate load transport was significantly lower from the City Stormwater on a per area basis. Load estimates at the watershed scale identified that cropland areas transported 57.6 times higher nitrate loads, 2.1 times higher TP loads, 2.3 times lower ammonium loads, and 2.6 times lower TSS loads than urban areas. Practices that reduce peak flow, and remove sediments and nutrients can help reduce the transport of these contaminants.

The Fertilized Field transported significantly higher nitrate load per unit area than the Unfertilized Field, whereas, the Unfertilized Field transported significantly higher loads of TSS and TP. Even though the Unfertilized Field did not receive fertilizer nor manure since 2005, the cumulative nitrate load per area was still 58% of that of the Fertilized Field. The significant decreasing trends for nitrate and TP concentrations from the Fertilized Field are a positive development; however, further investigation is needed to determine causes.

Assessment of contaminant transport dynamics showed an increase in concentration of TSS at high flows for the City Stormwater, nitrate and TP for the Fertilized Field, and TSS for the Unfertilized Field. For the City Stormwater, ammonium concentration reduced as flow increased. Hydrology (i.e., flow variation) was also a major factor in the dynamics of load transport, though it had a lesser role for TSS from the Fertilized and Unfertilized Fields. Therefore, contaminant removal during high flows should be targeted to achieve a substantial load transport reduction.

Furthermore, we demonstrated the importance of accounting for serial correlation in a paired *t*-test and linear regression analysis, and failure to account for this effect can provide misleading results. In summary, our findings provide new insight into the extent of contaminant transport from agricultural and urban landscapes that can be used in future modeling efforts to direct appropriate management practices to watersheds with farmland and urban areas in close proximity.

## Disclaimer

Mention of trade names or commercial products in this publication is solely for the purpose of providing specific information and does not imply recommendation or endorsement by the U.S. Department of Agriculture. USDA is an equal opportunity provider and employer.

## Supporting Information

S1 File Days that were used in comparisons. (PDF)

S2 File Durbin-Watson statistic and *p*-value for testing positive and negative autocorrelation. (PDF)

S3 File Crop yields for the Unfertilized and Fertilized Fields. (PDF)

S4 File Relationship between daily nitrate load and daily flow depth. (PDF)

S5 File Relationship between daily nitrate concentration and daily flow depth. (PDF)

S6 File Coefficient of determination for the linear regression. (PDF)

S7 File KMZ of the study area. (RAR)

S1 Fig Crop residue of the Unfertilized Field on May 4, 2010. (JPG)

S2 Fig Crop residue of the Fertilized Field on May 5, 2010. (JPG)

S1 Multimedia City Stormwater time-lapse on May 22, 2010. (MP4)

S1 Appendix Comparison Fertilized and Unfertilized Field data. (XLSX)

S2 Appendix Comparison City Stormwater and Fertilized Field data. (XLSX)

S3 Appendix Transport dynamic data. (XLSX)
