# Supplementary material for: Comparison of Contaminant Transport in Agricultural Drainage Water and Urban Stormwater Runoff
Source: PLoS One. 2016 Dec 8;11(12):e0167834. doi: 10.1371/journal.pone.0167834 (PMC5145188; doi:10.1371/journal.pone.0167834)
Supplement: S1 File — (PDF) [file pone.0167834.s001.pdf]

Comparison of contaminant transport in agricultural drainage water and urban stormwater runoff

Ehsan Ghane, Andry Z. Ranaivoson, Gary W. Feyereisen, Carl J. Rosen, John F. Moncrief

S1 File

Table 1
Days that were used when comparing City Stormwater and Fertilized Field. Years 2008 and 2013 were excluded.

	Year				
	2007	2009	2010	2011	2012
Sample size (n)	121	147	214	144	166
Dates	1 Jun to 21 Sep<br>2 Oct to 9 Oct	1 May to 13 Jul<br>20 Aug to 31 Oct	1 Apr to 31 Oct	1 Apr to 22 Aug	1 Apr to 9 Jul<br>31 July<br>28 Aug to 31 Oct

Table 2
Days that were used when comparing Unfertilized and Fertilized Field.

	Year						
	2007	2008	2009	2010	2011	2012	2013
Sample size (n)	144	103	184	214	133	193	100
Dates	1 Apr to 8 Oct<br>17 Oct to 31 Oct	20 Apr to 31 Jul	1 May to 31 Oct	1 Apr to 31 Oct	1 Apr to 11 Aug	1 Apr to 9 Jul<br>31 Jul to 31 Oct	1 Apr to 9 Jul
