# Supplementary material for: Comparison of Contaminant Transport in Agricultural Drainage Water and Urban Stormwater Runoff
Source: PLoS One. 2016 Dec 8;11(12):e0167834. doi: 10.1371/journal.pone.0167834 (PMC5145188; doi:10.1371/journal.pone.0167834)
Supplement: S2 File — (PDF) [file pone.0167834.s002.pdf]

Comparison of contaminant transport in agricultural drainage water and urban stormwater runoff

Ehsan Ghane, Andry Z. Ranaivoson, Gary W. Feyereisen, Carl J. Rosen, John F. Moncrief

S2 File

Table 1

Durbin-Watson statistic and *p*-value for testing positive and negative autocorrelation when comparing Stormwater and Fertilized Field.

Description	Durbin-Watson statistic	Positive autocorrelation *p*-value	Negative autocorrelation *p*-value
Precipitation	2.37	1.000	<0.001
Flow depth	1.30	<0.001	1.000
Nitrate load	0.59	<0.001	1.000
Ammonium load	0.84	<0.001	1.000
Total suspended solids load	1.39	<0.001	1.000
Total phosphorus load	1.24	<0.001	1.000

Table 2

Durbin-Watson statistic and *p*-value for testing for positive and negative autocorrelation when comparing Unfertilized and Fertilized Fields.

Description	Durbin-Watson statistic	Positive autocorrelation *p*-value	Negative autocorrelation *p*-value
Flow depth	1.01	<0.001	0.016
Nitrate load	0.77	<0.001	1.000
Ammonium load	0.82	<0.001	1.000
Total suspended solids load	1.39	<0.001	1.000
Total phosphorus load	0.86	<0.001	1.000
