# Supplementary material for: Comparison of Contaminant Transport in Agricultural Drainage Water and Urban Stormwater Runoff
Source: PLoS One. 2016 Dec 8;11(12):e0167834. doi: 10.1371/journal.pone.0167834 (PMC5145188; doi:10.1371/journal.pone.0167834)
Supplement: S3 File — (PDF) [file pone.0167834.s003.pdf]

Comparison of contaminant transport in agricultural drainage water and urban stormwater runoff

Ehsan Ghane, Andry Z. Ranaivoson, Gary W. Feyereisen, Carl J. Rosen, John F. Moncrief

S3 File

Table 1
Crop yields for the Unfertilized and Fertilized Fields.

Crop	Year	Grain yield (Mg ha^{-1})	
		Unfertilized Field	Fertilized Field
Soybean	2007	3.52	3.50
	2010	4.14	3.36
	Average	3.83	3.43
Corn	2008	8.79	12.31
	2009	6.84	13.07
	2011	8.10	9.22
	2012	6.84	11.74
	2013	4.52	10.55
	Average	7.02	11.38
