# Supplementary material for: Comparison of Contaminant Transport in Agricultural Drainage Water and Urban Stormwater Runoff
Source: PLoS One. 2016 Dec 8;11(12):e0167834. doi: 10.1371/journal.pone.0167834 (PMC5145188; doi:10.1371/journal.pone.0167834)
Supplement: S4 File — (PDF) [file pone.0167834.s004.pdf]

Comparison of contaminant transport in agricultural drainage water and urban stormwater runoff

Ehsan Ghane, Andry Z. Ranaivoson, Gary W. Feyereisen, Carl J. Rosen, John F. Moncrief

S4 File

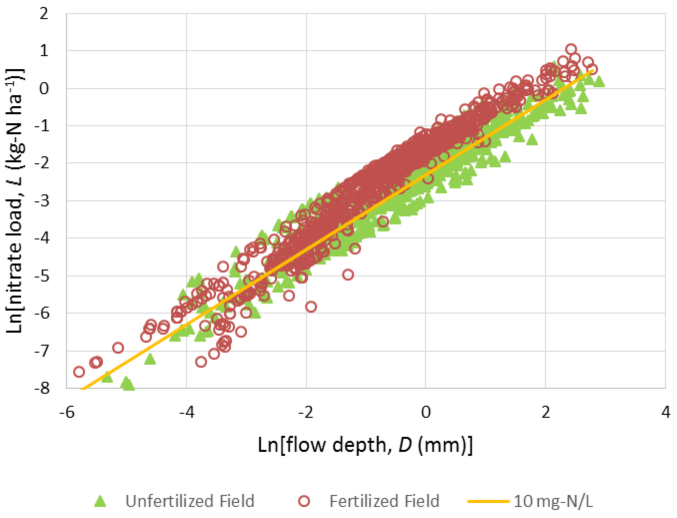

Fig. 1. Relationship between daily nitrate load and daily flow depth over the period of the study for Fertilized Field (n=907) and Unfertilized Field (n=663). The line represents the loads at constant nitrate concentration of 10 mg-N L^{-1}.
