# Supplementary material for: Comparison of Contaminant Transport in Agricultural Drainage Water and Urban Stormwater Runoff
Source: PLoS One. 2016 Dec 8;11(12):e0167834. doi: 10.1371/journal.pone.0167834 (PMC5145188; doi:10.1371/journal.pone.0167834)
Supplement: S5 File — (PDF) [file pone.0167834.s005.pdf]

Comparison of contaminant transport in agricultural drainage water and urban stormwater runoff

Ehsan Ghane, Andry Z. Ranaivoson, Gary W. Feyereisen, Carl J. Rosen, John F. Moncrief

S5 File

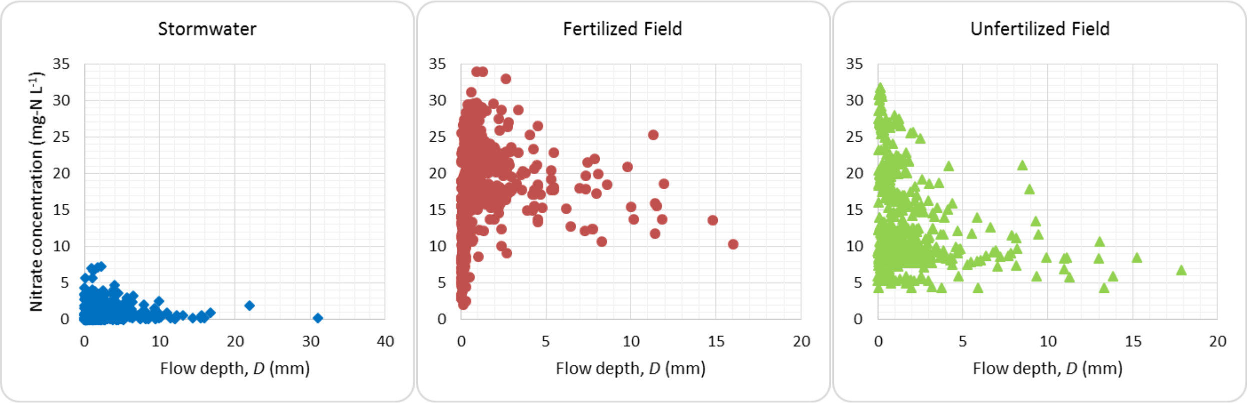

Fig. 1. Relationship between daily nitrate concentration and daily flow depth.

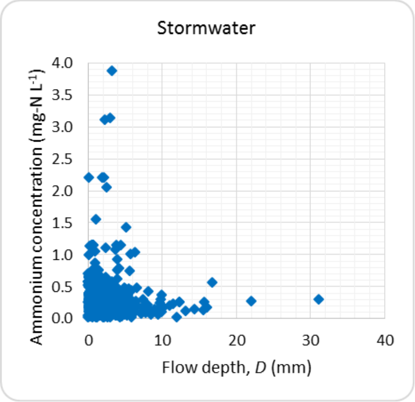

Fig. 2. Relationship between daily ammonium concentration and daily flow depth.

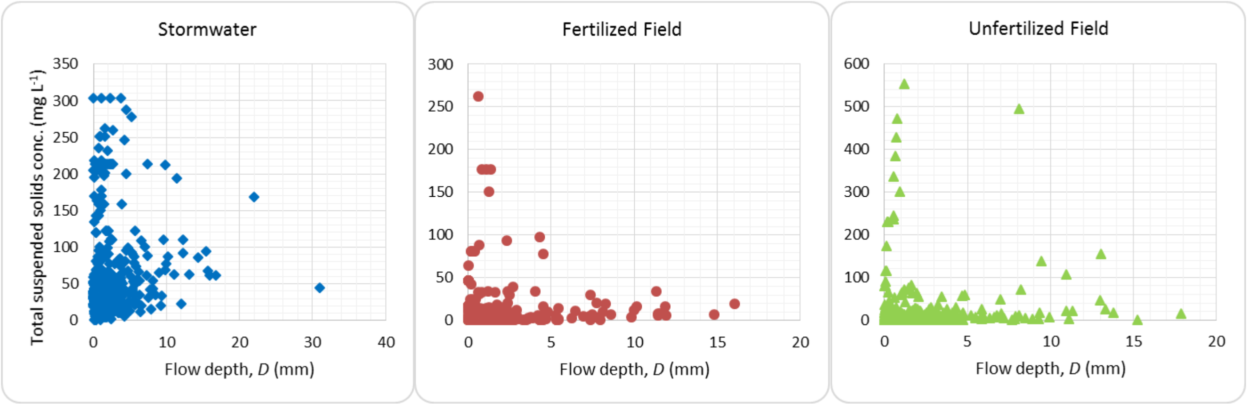

Fig. 3. Relationship between daily total suspended solids concentration and daily flow depth.

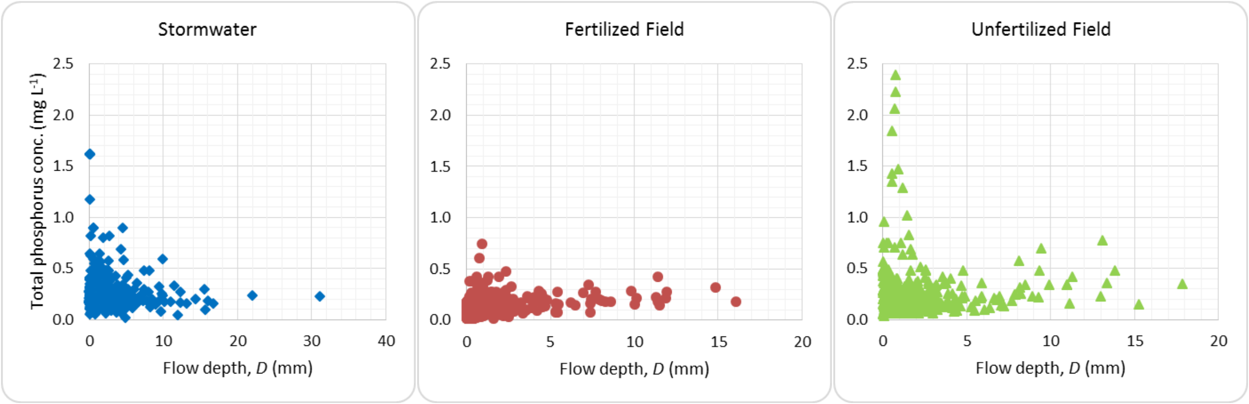

Fig. 4. Relationship between daily total phosphorus concentration and daily flow depth.
