# Supplementary material for: Comparison of Contaminant Transport in Agricultural Drainage Water and Urban Stormwater Runoff
Source: PLoS One. 2016 Dec 8;11(12):e0167834. doi: 10.1371/journal.pone.0167834 (PMC5145188; doi:10.1371/journal.pone.0167834)
Supplement: S6 File — (PDF) [file pone.0167834.s006.pdf]

Comparison of contaminant transport in agricultural drainage water and urban stormwater runoff

Ehsan Ghane, Andry Z. Ranaivoson, Gary W. Feyereisen, Carl J. Rosen, John F. Moncrief

S6 File

Table 1
Coefficient of determination for the linear regression of natural log of concentration versus natural log of flow depth over the entire period of the study.

Site	Coefficient of determination, R^2	
	Ordinary least squares estimate	Adjusted for serial correlation
Nitrate		
Stormwater	0.02	<0.01
East Field	0.20	0.08
Unfertilized Field	0.02	0.02
Ammonium		
Stormwater	0.02	0.01
Total suspended solids		
Stormwater	0.01	0.02
East Field	<0.01	<0.01
Unfertilized Field	<0.01	<0.01
Total phosphorus		
Stormwater	0.03	<0.01
East Field	0.16	0.03
Unfertilized Field	<0.01	<0.01

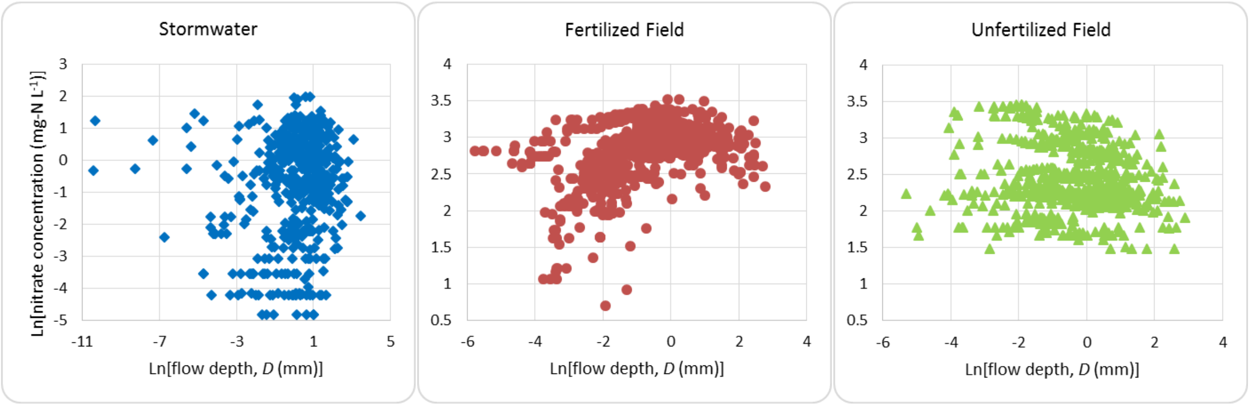

Fig. 1. Relationship between natural log of daily nitrate concentration and natural log of daily flow depth.

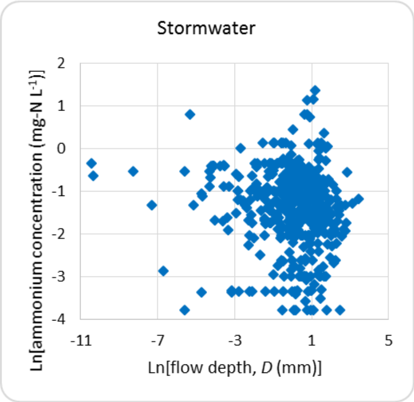

Fig. 2. Relationship between natural log of daily ammonium concentration and natural log of daily flow depth.

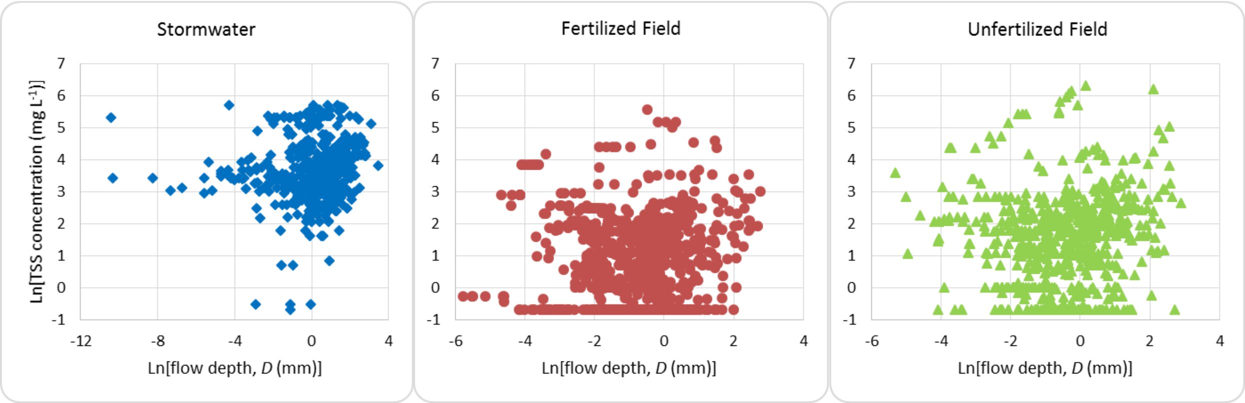

Fig. 3. Relationship between natural log of daily total suspended solids (TSS) concentration and natural log of daily flow depth.

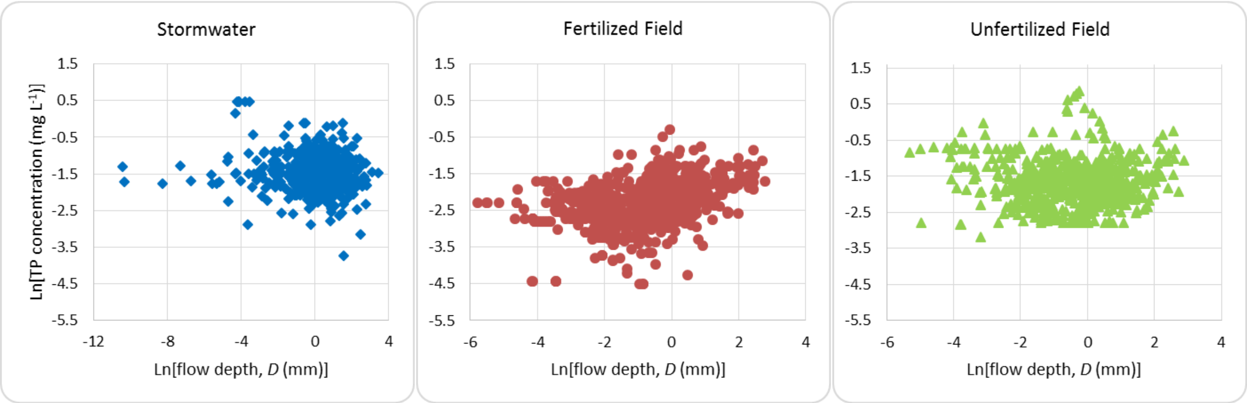

Fig. 4. Relationship between natural log of daily total phosphorus (TP) concentration and natural log of daily flow depth.
