# Supplementary figures and images for: Comparison of Contaminant Transport in Agricultural Drainage Water and Urban Stormwater Runoff
Source: PLoS One. 2016 Dec 8;11(12):e0167834. doi: 10.1371/journal.pone.0167834 (PMC5145188; doi:10.1371/journal.pone.0167834)

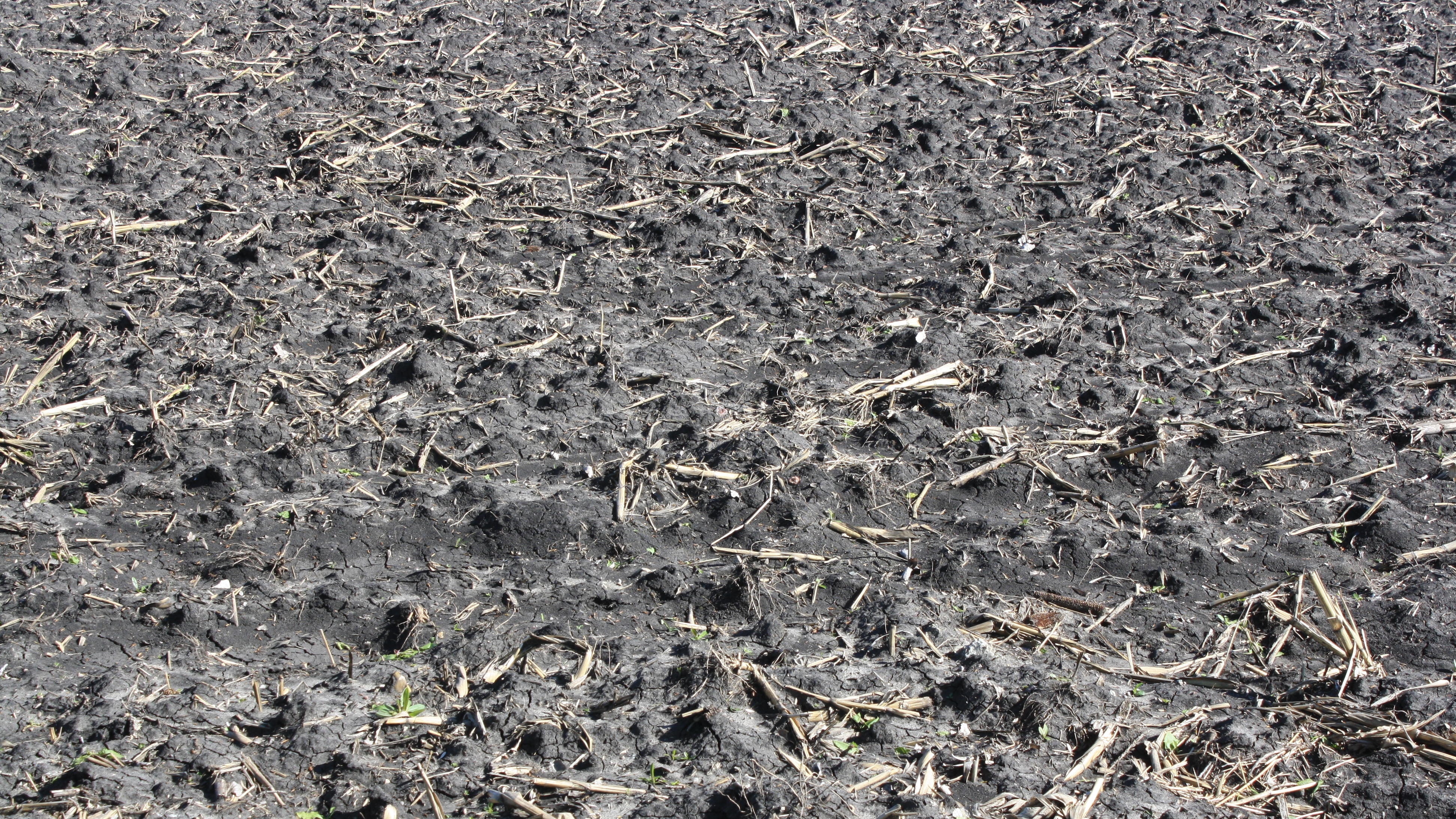

Supplement: S1 Fig — (JPG) [file pone.0167834.s008.JPG]

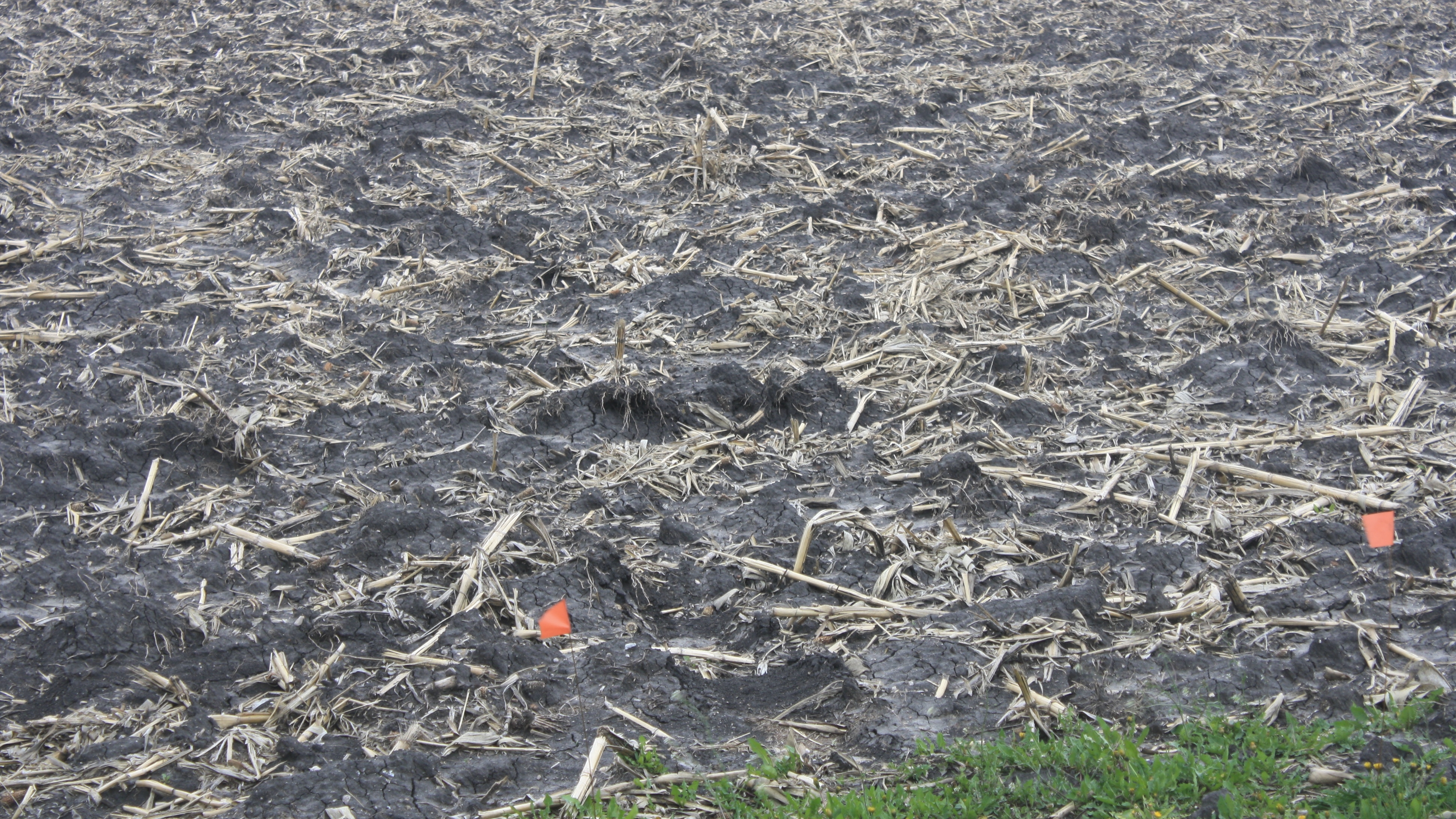

Supplement: S2 Fig — (JPG) [file pone.0167834.s009.JPG]
